# Major depression associated with a levonorgestrel-releasing intrauterine system mimicking frontotemporal dementia: a case report

**DOI:** 10.3389/fpsyt.2023.1266419

**Published:** 2023-09-13

**Authors:** Valeria Valencia-Cifuentes, Carlos A. Cañas, Juan Carlos Rivas

**Affiliations:** ^1^Department of Neurology, Fundación Valle del Lili, Cali, Colombia; ^2^Facultad de Ciencias de la Salud, Universidad Icesi, Cali, Colombia; ^3^Universidad ICESI, CIRAT: Centro de Investigación en Reumatología, Autoinmunidad y Medicina Traslacional, Cali, Colombia; ^4^Department of Rheumatology, Fundación Valle del Lili, Cali, Colombia; ^5^Department of Psychiatry, Fundación Valle del Lili, Cali, Colombia; ^6^Department of Psychiatry, Universidad del Valle, Cali, Colombia; ^7^Hospital Departamental Psiquiátrico, Universitario del Valle, Cali, Colombia

**Keywords:** anxiety, depressive cognitive disorder, contraception, hormonal contraceptive, progestin-only contraceptive, adverse event

## Abstract

This case illustrates the adverse cognitive and affective effects associated with the use of an intrauterine hormonal contraceptive, which could be confused with symptoms of early onset dementia. We present a case of a 42-year-old woman diagnosed with seronegative spondyloarthropathy who subsequently developed anxiety and depressive symptoms after the implantation of a Levonorgestrel-Releasing Intrauterine System (LNG-IUS). Three years later, she began to experience memory and attentional failures, refractory pain, and severe depression. The progression of psychiatric symptoms led to a diagnosis of bipolar affective disorder and treatment with antidepressants and anxiolytics. Due to cognitive and psychiatric symptoms, autoimmune encephalitis was considered, but no improvement was shown with treatment. Early onset dementia was suspected, and a brain PET scan revealed frontal lobe hypometabolism. An adverse effect of LNG-IUS was considered; after its removal, mood and cognitive function improvements were observed. This case report emphasizes the importance of considering organic causes of unexplained psychiatric manifestations and highlights the potential impact of hormonal interventions on mental health.

## 1. Introduction

In recent decades, hormonal contraceptives such as oral contraceptives, hormonal patches, injections, and Intrauterine Devices (IUDs) have become widely available, and usage has increased (1). The Levonorgestrel-Releasing Intrauterine delivery system (LNG-IUS) is an effective reversible contraceptive device lasting up to 5 years that can be used to treat several gynecological conditions. The most frequent adverse drug reactions of LNG-IUS are menstrual irregularities and changes in bleeding patterns; however, it has been associated with various undesirable side effects, such as ovarian cysts, acne, weight gain, depression, and decreased libido (2). Therefore, alongside the undeniable benefits and safety of such devices, concerns have arisen regarding the potential impact of these contraceptives on mood disorders, particularly the development of depression (3–6).

Depression is a debilitating mental health disorder characterized by persistent depressed mood, diminished interests, and/or impaired cognitive function that causes significant impairments in social, work, or personal life (7). Approximately 280 million people worldwide have depression, an estimated 5% of the adult population, being more common in women (8). Cognitive and functional impairments imitating neurodegenerative diseases may be associated with depression; this condition was described in the 1980s as a depressive cognitive disorder and has been incorporated as part of the reversible and treatable forms of dementia (9). Depression with cognitive impairments implies incipient dementia and merits diagnostic workup.

According to the Diagnostic and Statistical Manual of Mental Disorders, fifth edition (DSM-V), a major neurocognitive disorder is defined as the presence of significant acquired cognitive impairment in one or more cognitive domains that represents a substantial decline from baseline function and reduces independence in daily activities (10). This disorder results from a progressive process during which patients initially experience an insidious decline in normal cognition and/or social behavior (11). Early onset dementia usually refers to cases of dementia in adults before the age of 65 years, and it is often misdiagnosed due to nonspecific symptoms and findings (12).

We describe a case of a 42-year-old woman who developed substantial cognitive impairment, with neuropsychological and brain PET-CT findings suggestive of frontotemporal dementia secondary to a severe depressive syndrome 1 year after insertion of an LNG-IUS. These symptoms improved after the removal of the device.

## 2. Case presentation

A 42-year-old right-handed female electronic engineer was asymptomatic until the age of 28 years when she started experiencing skeletal pain and progressive depressive symptoms. Initially, the patient was evaluated by a rheumatologist and diagnosed with fibromyalgia. Analgesics were prescribed, resulting in partial improvement in pain. Seeking further evaluation, she consulted a psychiatrist. Her symptoms were considered suggestive of bipolar affective disorder type 1, and treatment with antidepressants and anxiolytics was initiated.

When she was 37 years old, after a pregnancy, she decided to discontinue oral contraceptives, and an LNG-IUS was inserted as a contraceptive method. A year later, she noticed work-related problems, argued frequently, was irritable, withdrawn from family and close friends, no longer enjoyed activities, forgot appointments, and had trouble focusing on activities and understanding what she read. She started to experience apathy and lack of motivation, neglecting her daughter, and losing interest in household chores. Her pain worsened, leading to several hospitalizations and cycles of intravenous ketamine and muscle blocks with poor response. Prior to the patient’s pregnancy, she was using oral contraceptives containing drospirenone and ethinyl estradiol. There did not appear to be a relationship between it and depressive symptoms.

At 39, she was diagnosed with seronegative spondyloarthropathy and started treatment, experiencing partial pain improvement. However, the psychomotor symptoms accelerated, and anxiety symptoms increased. Laboratory testing, lumbar puncture, neuropsychological assessments, and structural neuroimaging were performed. Metabolic and infectious diseases were ruled out. Brain MRI with contrast was normal. Neuropsychological assessment revealed a mild frontal predominance of cognitive impairment, anxiety, and severe depression. In our efforts to identify organic causes for the cognitive impairments, we conducted an extensive battery of laboratory tests and brain imaging. She was hospitalized and found negative for a panel of autoantibodies, including anti-α-amino-3-hydroxy-5-methyl-4-isoxazolepropionic acid receptor (anti-NMDAR). The onset and progression of symptoms prompted consideration of various potential causes, including autoimmune encephalitis and early onset dementia. Despite the absence of brain MRI abnormalities, and negative anti-NMDA antibodies, a decision was made to pursue management with plasmapheresis based on the criteria for probable antibody-negative autoimmune encephalitis (13). The patient underwent six sessions of plasmapheresis but did not improve.

**Figure 1:**
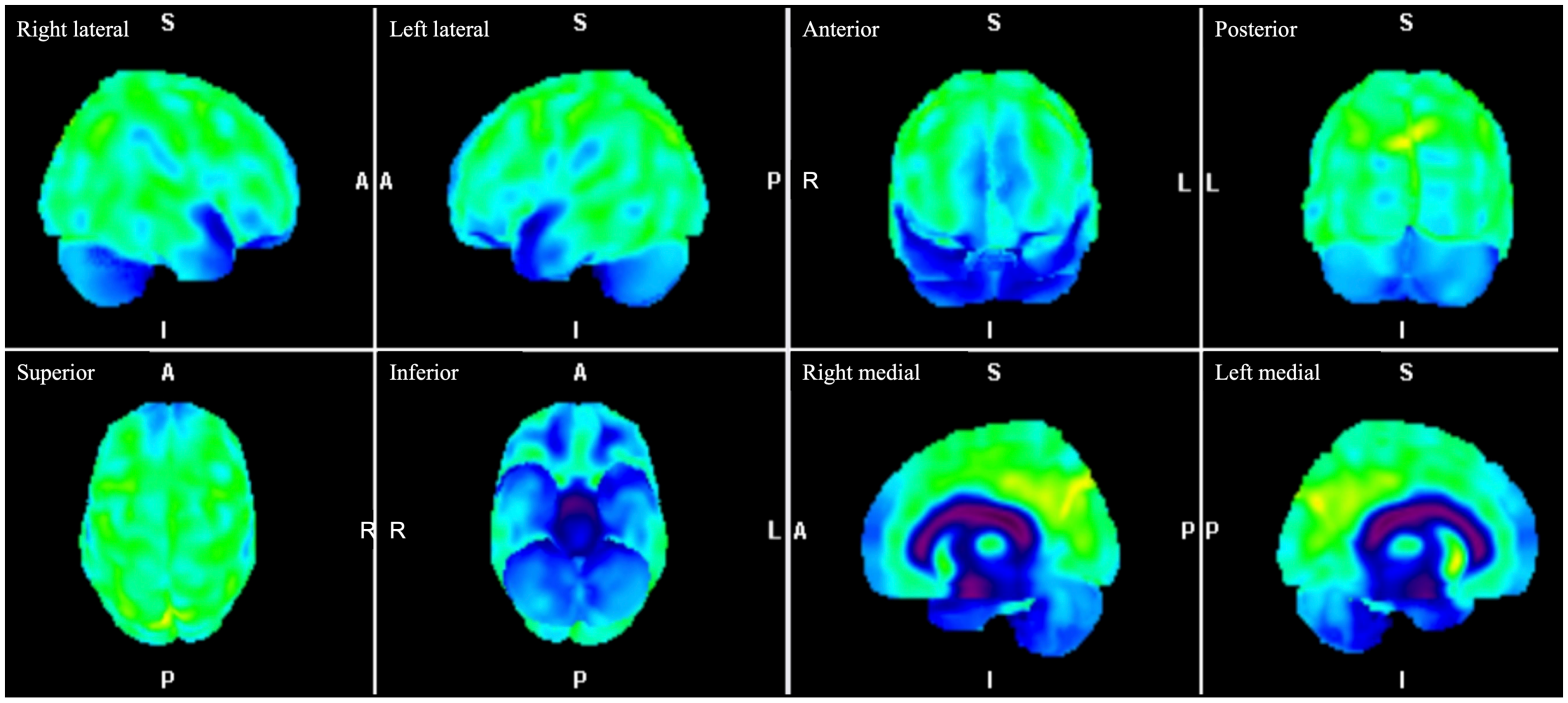
Brain ^18^F-FDG PET images. Three-dimensional stereotactic surface projection images of the right lateral, left lateral, anterior, posterior, superior, inferior, right medial and left medial projections demonstrate hypometabolism in the frontal lobes, with greater hypometabolism toward the superior orbital gyrus and inferior opercular, triangular, and opercular gyri, with less involvement of the temporal lobe. ^18^F-FDG PET, fluorine-18 fluorodeoxyglucose positron emission tomography.

As symptoms progressed, the possibility of early onset dementia was considered. Upon review of the International Consensus Criteria for Behavioral Variant Frontotemporal Dementia (bvFTD), physicians determined that the patient fulfilled the criteria for possible bvFTD (12). The patient did not exhibit any dementia risk factor, including traumatic injury or a history of alcohol or drug abuse, nor did she have a family history of dementia. Furthermore, neurological examination revealed the absence of pyramidal, extrapyramidal, cerebellar of peripheral nerve involvement. A second neuropsychological assessment revealed deterioration in some cognitive domains, such as visual and verbal memory, comprehension, and executive functions, without improvement in anxiety or depressive symptoms. Frontotemporal dementia was suspected, and a brain ^18^F-FDG PET scan was performed, revealing hypometabolism in the frontal lobes, with more significant hypometabolism toward the superior orbital gyrus and inferior opercular, triangular, and opercular gyri, with less involvement of the temporal lobe (Figure 1). These findings were consistent with a neurodegenerative process with frontal predominance.

The case was discussed at a multidisciplinary medical board meeting, and attention was drawn to the late onset of the psychiatric symptoms and the progressive deterioration, with fluctuating symptoms. The possibility of a dementia was ruled out. It was suggested that further investigation be conducted to explore the possible relationship between contraceptive methods and the described changes. The LNG-IUS device was considered the likely cause of the patient’s symptoms and was removed.

Two weeks after the device was removed, a significant recovery of mood was noticed, with improvement in her functional capacities. A neuropsychological evaluation conducted 6 months after the LNG-IUS removal noted improved attention, memory, and verbal fluency. She scored higher on Addenbrooke’s cognitive examination, the Mini-Mental State Examination, and the Montreal Cognitive Assessment in the cognitive assessment. Regarding verbal memory, there was evidence of better performance in immediate memory and short-term recall. The patient’s subjective complaints about her memory decreased. She also improved her performance on tasks involving alternating attention, visual memory, and calculation. No significant behavioral alterations were reported, and she exhibited a lower score on the Beck Depression Inventory compared to those on the two previous evaluations. Likewise, the number of hospitalizations due to difficult-to-manage pain decreased. She has resumed employment and has a good relationship with her family, and her social life has recovered. Table 1 summarizes the essential changes documented in the neuropsychological evaluations.

**Table 1:** Neuropsychological results over time.

	Addenbrooke’s cognitive examination score	Frontal behavioral inventory score		Beck’s depression inventory score	
Initial October 2020	84/100	36/72	Frontal dysfunction	40/63	Severe depression
Follow-up 1 June 2021	72/100	55/72		46/63	
Follow-up 2 April 2020 (6 months after IUS removal)	88/100	26/72	No frontal dysfunction	28/63	Moderate depression

**Figure 2:**
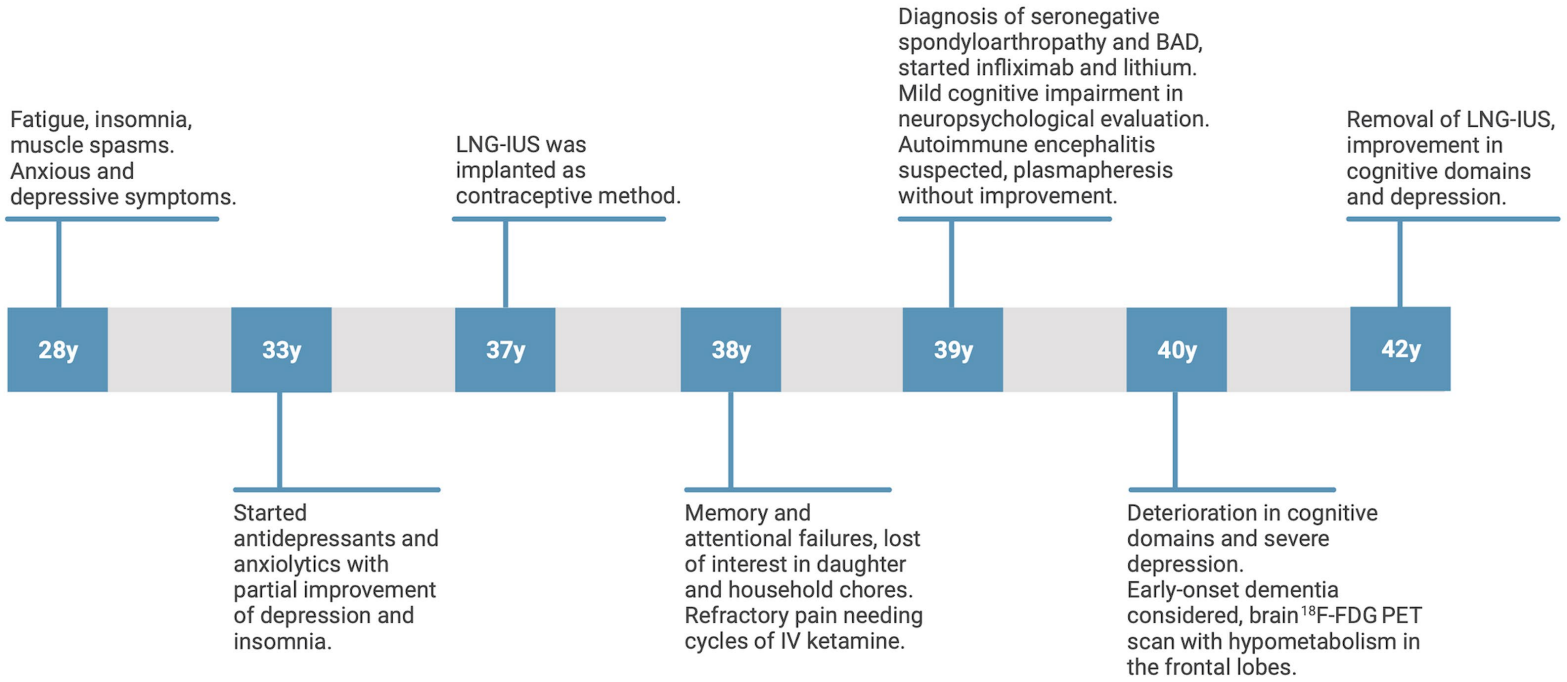
Timeline showing the course of the patient’s psychiatric symptoms, cognitive impairments, pharmacological treatment, differential diagnosis, and insertion and removal of the LNG-IUS over time. LNG-IUS, levonorgestrel-releasing intrauterine system. Illustration created with Biorender.com.

Figure 2 highlights the critical findings in the patient over time.

## 3. Discussion

In the present report, we describe a case of a woman who developed depressive and anxiety symptoms and cognitive decline after the insertion of an LNG-IUS. She fulfilled the criteria for bvFTD, and brain PET CT findings were consistent with a frontotemporal neurodegenerative process. After the removal of the device, psychiatric and cognitive symptoms improved significantly. As the patient was a woman of reproductive age who exhibited a prolonged progression of symptoms, depression being the most refractory symptom, the possibility of an adverse effect of her intrauterine contraceptive device was considered. After reviewing the Naranjo algorithm and the definitions of Edwards and Aronson, a causative role of the LNG-IUS in the development of cognitive decline and psychiatric symptoms in our patient was classified as “possible” (14, 15).

LNG-IUSs can potentially cause depression through various mechanisms involving the influence of estrogen and progesterone on neurochemistry and brain function. Several studies have highlighted the influence of sex steroid hormones, including estrogen and progesterone, on cortical and subcortical regions associated with emotional and cognitive processing (3, 16, 17). Estrogen receptors are mainly present in the hypothalamus, hippocampus, amygdala, and brainstem, whereas progesterone receptors are abundant in the amygdala, cerebellum, cortex, hippocampus, and hypothalamus (3). Estrogen is believed to have a neuroprotective effect on regions such as the hypothalamus, hippocampus, amygdala, and brainstem, safeguarding against neurodegenerative diseases, cognitive decline, and affective disorders. Functional brain imaging studies have demonstrated that estrogen regulates the activation of brain areas involved in emotional and cognitive processing, such as the amygdala and dorsolateral prefrontal cortex (17).

On the other hand, endogenous progesterone o progestogens does not possess those neuroprotective properties and may exacerbate mood symptoms. Plausible explanations for the relationship between progestogens and depression include the augmentation of GABA-induced inhibition of glutamate transmission and an increase in monoamine oxidase levels, leading to decreased serotonin concentrations (6). In addition, allopregnanolone and pregnanolone act as positive modulators of the GABA_A_ receptor, which typically have anxiolytic, sedative, and antiepileptic effects (18). However, despite these expected effects, it is noted that high concentrations of allopregnanolone are linked to negative mood changes during the menstrual cycle. This paradoxical effect is seen because substances that positively modulate the GABA_A_ receptor can induce opposite reactions in some individuals. These substances can lead to irritability, aggression, depression, confusion, violent behavior, and loss of impulse control in these individuals compared to a placebo (19). The interaction of progestogens metabolites with the γ-aminobutyric acid A receptor complex, a major inhibitory system in the central nervous system, is another likely mechanism (5).

So that, hormonal contraceptives downregulate endogenous hormone production, alter the cyclical fluctuations of endogenous hormones, and replace them with constant levels of potent synthetic estrogens and progestins. These processes could potentially impact brain regions that are particularly responsive to the effects of these synthetic hormones, including the hippocampus, amygdala, and prefrontal cortex (20). These brain areas play a role in functions like thinking and emotion. As for cognitive alterations, while using hormonal contraceptives, past research has produced largely inconclusive findings, primarily due to limited sample sizes and a lack of control over the specific contraceptive formulations being used (20, 21).

A Danish national registry study assessed the influence of specific hormonal contraceptives on the risk of the first use of antidepressants and first diagnosis of depression among inpatients and outpatients at a psychiatric hospital in over one million women. Women using LNG-IUSs had an RR of first using antidepressants 1.4 (16). The fact that the use of progestin-only products, including LNG-IUSs, increased the risk of using antidepressants and being diagnosed with depression supports the finding that even though the levonorgestrel intrauterine system primarily works locally, levonorgestrel reaches systemic circulation (1, 16). Likewise, a cohort study was performed to compare the incidence of adverse psychiatric events between groups of women who were new users of levonorgestrel-releasing and nonhormonal IUDs in UK general practice (4). The results showed a positive association between the prescription of an LNG-IUS and depression and a nonsignificant association of LNG-IUSs with anxiety and sleep problems in women without these conditions before the use of the IUD. In addition, Zeiss et al. (22) reported a case of a 41-year-old woman who developed depressive and anxiety symptoms after her second LNG-IUS. After a complete examination, a mental disorder was considered. The device was removed and led to the remission of her cognitive complaints. The patient expressed that she was never informed about the potential adverse effects of this device on mental health, which is why this case supports observations regarding mood changes in response to LNG-IUSs and emphasizes why psychiatric symptoms must be communicated as adverse drug reactions.

On the other hand, recent studies have found that hormonal contraceptive use does not increase depressive symptoms in adult women. A systematic review and network meta-analysis of randomized clinical trials compared women randomly assigned to receive any form of a hormonal contraceptive with women randomly assigned to receive any other form of a nonhormonal contraceptive or placebo. More than 5,000 patients were included, and 10 interventions were compared. Compared with the placebo group, the hormonal contraceptive group exhibited worsening depressive symptoms (23).

Cognitive symptoms are frequently observed in individuals with major depression, and in some cases, these impairments in higher cognitive function become the prominent feature, significantly impacting overall functioning (9). Manifestations of cognitive deficits in individuals with depression vary across individuals and are influenced by factors specific to the individual and their illness. The severity of cognitive deficits is proportional to the frequency and duration of depressive episodes (24). Furthermore, factors such as age, age at the onset of depression, level of education, depression subtype, inflammatory status, treatment regimen, and childhood adversity have been found to influence cognitive performance in individuals with depression (25).

Cognitive impairments in depression are associated with attention problems that impact memory performance. In our patient, the main cognitive alterations documented in the neuropsychological evaluations were in visual and verbal memory, comprehension, and executive functions. A systematic review that investigated the extent of cognitive impairment in depression in the acute and remitted states showed that depressed patients present deficits in processing speed, learning, and memory and impairments in attention, inhibition, verbal fluency, and working memory (26). Moreover, despite the remission of the depressive symptoms, deficits in attention, learning, and memory, and working memory persisted.

Early onset dementia is a frequently misdiagnosed condition. This can be attributed to the presence of multiple differential diagnoses, such as Alzheimer’s disease, vascular dementia, frontotemporal lobar degeneration, traumatic head injury, alcohol-related dementia, among others. Patients exhibit cognitive deficits beyond memory loss and neuropsychiatric features that appear disproportionate to any cognitive impairments (27). Diagnosis and classification of neurocognitive disorders is difficult in the early stages of the disease, and the use of certain medications and comorbid conditions such as depression makes the diagnosis even more challenging. Therefore, neuropsychological testing and neuroimaging are necessary to evaluate individuals with mild cognitive impairment and dementia. Frontotemporal dementia is a group of clinical syndromes caused by a neurodegenerative disease characterized by progressive changes in behavior, language, or executive function. In a 2020 study of patients with bvFTD, a diagnosis was not established for more than 1 year in over 40% of patients, and the initial diagnosis was depression or another psychiatric condition in more than 20% of patients (28).

Positron emission tomography with FDG is a beneficial imaging modality for these disorders; the use of FDG allows imaging and measurement of cerebral glucose metabolism, providing information on synaptic and neural function (29). Distinctive metabolic patterns observed in FDG PET scans can significantly enhance the accuracy of clinical diagnosis for specific types of dementia, including frontotemporal dementia, Alzheimer’s disease, and Lewy bodies dementia (30). Each of these dementia types exhibits unique metabolic signatures, although there can be some overlap. Furthermore, a normal or preserved cerebral uptake pattern can help differentiate reversible pseudodementia caused by depression from a primary neurodegenerative syndrome (31). The cingulate gyrus is among the regions affected early in many neurodegenerative disorders (30). FTD is characterized by reduced metabolism in the frontal and anterior temporal lobes, with the involvement of the anterior cingulate gyrus. When patients show no structural abnormalities, such as our patient, the possibility of false-positive findings in FDG PET scans for dementia is low; nevertheless, disorders such as depression or autoimmune encephalitis can present such findings (32, 33). Some PET studies have shown reduced lateral prefrontal metabolism and increased medial prefrontal and subgenual cingulate metabolism in depressed patients (34). Kotagal et al. (26) also reported a patient with peripartum antibody-mediated voltage-gated potassium channel encephalitis whose FDG PET findings suggest a frontotemporal dementia-like disorder.

This case demonstrates that cognitive impairment associated with severe depression might be another crucial adverse drug reaction to LNG-IUSs. While depression is a recognized side effect, no previous reports have documented a case of reversible dementia. It required a thorough medical history, imaging studies, neuropsychological tests, and a multidisciplinary team to reach a correct diagnosis. The uniqueness and rarity of this case lie in its atypical presentation, the complexity of its differential, the impact of hormonal intervention, and the insights it offers into the potential interplay between hormonal changes, psychiatric symptoms, and cognitive function. It is essential to describe the potential occurrence of psychiatric symptoms in patients and highlight this risk for gynecologists and psychiatrists, as they may encounter more complex depressive symptoms that manifest in atypical ways, as in our patient. In such instances, conducting imaging and neuropsychological assessments is essential to enable timely diagnosis and ensure appropriate patient follow-up.

## Patients’ perspective

“After the removal of the IUS, my life changed. I have returned to being the woman I have always been.”

## Data availability statement

The original contributions presented in the study are included in the article/supplementary material, further inquiries can be directed to the corresponding author.

## Ethics statement

The studies involving humans were approved by Ethics Committee of Fundación Valle del Lili, Colombia. The studies were conducted in accordance with the local legislation and institutional requirements. The participants provided their written informed consent to participate in this study. Written informed consent was obtained from the individual(s) for the publication of any potentially identifiable images or data included in this article.

## Author contributions

VV-C: Conceptualization, Data curation, Investigation, Writing – original draft, Writing – review & editing. CAC: Supervision, Validation, Writing – review & editing. JCR: Supervision, Validation, Writing – original draft, Writing – review & editing.

## Funding

The author(s) declare that no financial support was received for the research, authorship, and/or publication of this article.

## Conflict of interest

The authors declare that the research was conducted in the absence of any commercial or financial relationships that could be construed as a potential conflict of interest.

## Publisher’s note

All claims expressed in this article are solely those of the authors and do not necessarily represent those of their affiliated organizations, or those of the publisher, the editors and the reviewers. Any product that may be evaluated in this article, or claim that may be made by its manufacturer, is not guaranteed or endorsed by the publisher.
